# Delayed filling of the superficial middle cerebral vein in acute large artery occlusion

**DOI:** 10.3389/fneur.2022.955804

**Published:** 2022-10-11

**Authors:** Jingsi Xu, Zheyu Zhang, Bo Jin, Yu Geng, Longting Lin, Sheng Zhang

**Affiliations:** ^1^The Second Clinical Medical College, Zhejiang Chinese Medical University, Hangzhou, China; ^2^Department of Neurology, People's Hospital of Hangzhou Medical College, Zhejiang Provincial People's Hospital, Hangzhou, China; ^3^Department of Neurology, John Hunter Hospital, Hunter Medical Research Institute, University of Newcastle, Newcastle, NSW, Australia

**Keywords:** large vessel occlusion, reperfusion therapy, superficial middle cerebral vein, prognosis, stroke

## Abstract

**Objective:**

This study aimed to determine whether baseline delayed filling of the superficial middle cerebral vein (SMCV) was an independent cause of stroke prognosis in patients with acute anterior large vessel occlusion (LVO).

**Methods:**

Consecutive patients with acute LVO [middle cerebral artery M1 ± intracranial internal carotid artery (ICA)] between March 2019 and May 2020 were included. Delayed SMCV was defined as delayed filling of SMCV in the affected side compared with the normal side throughout the venous phase on four-dimensional computed tomographic angiography (4D-CTA) reconstructed from CT perfusion imaging. The modified Rankin scale (mRS) was used to evaluate the prognosis of these patients 3 months after stroke.

**Results:**

Of 54 patients in total, 47 (87.0%) patients presented with baseline delayed SMCV, and 36 (76.6%) patients achieved SMCV reversal (ipsilateral delayed SMCV reversed to bilateral symmetrical SMCV) after reperfusion therapy. Successful reperfusion was independently associated with SMCV reversal [odds ratio (OR) = 69.328, 95% confidence interval (CI) = 2.818–175.342]. A significant association between baseline SMCV delay and a 3-month poor outcome (OR = 19.623, 95% CI = 1.567–245.727, *p* = 0.021) was observed using a multivariable regression analysis. Compared with patients with persistent delayed SMCV, patients with reversed SMCV did not show a significant difference in the risk of a 3-month poor outcome (OR = 1.177, 95% CI = 0.147–9.448).

**Conclusions:**

In patients with acute LVO, baseline delayed SMCV was an independent cause of poor stroke prognosis, and SMCV reversal cannot reverse the 3-month stroke prognosis. Therefore, the evaluation of baseline SMCV filling status should be strengthened in clinical practice.

## Introduction

In patients with stroke, cerebral venous filling deficit is caused by microvascular thrombosis after a decline in acute cerebral blood flow (1, 2). Cerebral venous filling deficit of the affected hemisphere (3, 4), especially the superficial middle cerebral vein (SMCV) (5–7), was noted to be associated with poor prognosis after stroke. However, whether this SMCV filling deficit is an independent cause of poor prognosis or just a secondary result of cerebral ischemia after acute arterial occlusion remains elusive.

In our previous study, we found that the absent filling of ipsilateral SMCV (abbreviated as SMCV-) could be reversed to normal (abbreviated as SMCV reversal) after reperfusion (5). However, our previous research had two main limitations. Firstly, the majority of the population included in that study were non-large artery occlusion. Chances are that SMCV- is uncommon in patients with non-large artery occlusion as SMCV- is related to the extent of hypoperfusion. Secondly, our previous methodology failed to distinguish congenital SMCV hypoplasia from stroke-induced SMCV filling deficit. To overcome these limitations, we included patients with large vessel occlusion (LVO) in this study and observed SMCV filling throughout the whole venous phase to better evaluate the relationship between dynamic changes of SMCV filling and reperfusion, and their impacts on stroke prognosis.

In this study, we hypothesized that the prognosis of patients with SMCV reversal could not reverse the poor prognosis for patients with baseline delayed SMCV, and the SMCV filling deficit at baseline should be the primary focus in the evaluation of stroke prognosis. The potential clinical significance of this study is that if the reversed filling of SMCV appears due to reperfusion and has no independent effect on clinical outcomes, the evaluation of baseline SMCV filling status will need to be strengthened in clinical practice. If the reversed SMCV filling can reverse the poor prognosis, this SMCV reversal may be used as an imaging marker for the outcome of reperfusion treatment in the future and become a potential target for intervention during reperfusion treatment.

## Methods

### Ethics statement

All patients or appropriate family members gave written informed consent before the study. The protocols of the study were approved by the human Ethics Committee of Zhejiang Provincial People's Hospital. All clinical investigations were conducted according to the principles set in the Declaration of Helsinki.

### Study population

We reviewed our consecutively collected data of patients with acute anterior LVO who were admitted within 24 h after stroke onset, which were registered into the International Stroke Perfusion Imaging Registry (INSPIRE) database from March 2018 to May 2019. Patients were enrolled in this study if they had (i) middle cerebral artery M1 segment and/or intracranial internal carotid artery (ICA) occlusion on pretreatment computed tomographic angiography (CTA), (ii) pre-stroke modified Rankin Scale (mRS) score, and (iii) 3–5 day–computer tomography perfusion (CTP) after admission. Patients were excluded if they had (i) bilateral or posterior acute ischemic lesions, (ii) poor image quality due to motion artifact, and (iii) difficulty to finish repetitive clinical or imaging assessments within a 90-day follow-up.

Eligible patients received intravenous thrombolysis prior to endovascular therapy (EVT). In routine clinical practice, EVT is offered to patients with (i) the presence of an LVO that is potentially retrievable, (ii) reasonable pre-stroke function (mRS 0–2), and (iii) limited medical comorbidity. The National Institute of Health Stroke Severity (NIHSS), the primary index for stroke severity, was assessed immediately before treatment and 24-h imaging. All patients were assessed at 90-day post-stroke onset for the functional outcome using the mRS.

### Imaging protocols

All patients underwent baseline NCCT and CTP, 24-h NCCT, and 3–5-day CTP after admission. All CT imaging scans were acquired on a 320-detector row 640-slice cone beam MDCT scanner (Aquilion One, Toshiba Medical Systems). Whole-brain NCCT was performed in one rotation (detector width, 16 cm). After NCCT, a CTP was acquired after administration of 50 ml of a contrast agent (Ultravist 370; Bayer HealthCare, Berlin, Germany) injected intravenously at a rate of 6 ml/s chased by 50 ml of saline (acquisition parameters: 120 kV, 128 mAs; scanning coverage = 240 mm, scanning width = 5 mm). Starting 7 s after contrast injection, a pulsed full-rotation scan with 18 time points acquired over 60 s with a total pulse image acquisition time of 9.5 s was used. The scanning protocol of follow-up NCCT was the same as that of baseline NCCT.

### Filling deficit of ipsilateral SMCV

We assessed SMCV filling on four-dimensional digital subtraction angiography (4D-DSA) automatically reconstructed from CTP using commercial software (MIStar; Apollo Medical Imaging Technology, Melbourne, Australia). For patients with an obvious head movement or head tilt that affected the quality of 4D-DSA, we used Vitrea fX (Version 1.0, Vital Images, Minnetonka, MN, USA) to assess SMCV filling.

**Figure 1:**
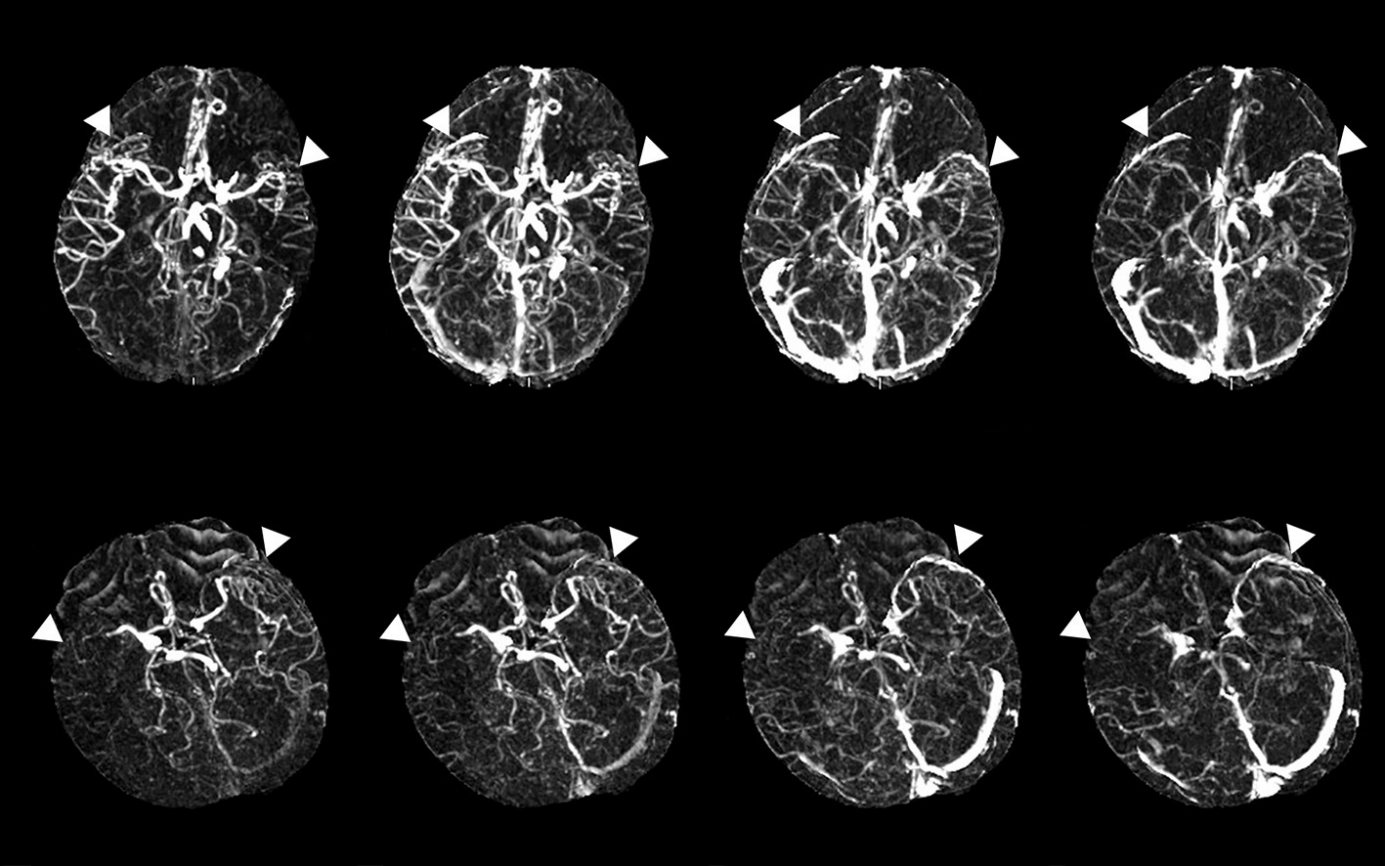
Examples of delayed superficial middle cerebral vein (SMCV) **(the upper line)** and absent filling of the ipsilateral superficial middle cerebral vein (SMCV-) **(the lower line)**. White triangles point to the SMCV.

The evaluation of the filling deficit of SMCV involved two types: delayed SMCV and absent SMCV. We defined delayed SMCV as the presence of SMCV filling at the affected side occurring later than that at the contralateral side. Patients who showed absent SMCV filling throughout the arterial phase to the venous phase were marked as absent SMCV (abbreviated as SMCV-) (5) (Figure 1).

In a follow-up imaging scan, we hypothesized that the opacification of SMCV had three types of outcomes:

1) Symmetric SMCV: symmetric SMCV of both sides from baseline to follow-up.
2) Reversed SMCV: ipsilateral delayed SMCV reversed to symmetric SMCV filling of both sides.
3) Persistent delayed SMCV: ipsilateral delayed SMCV sustained as ipsilateral delayed SMCV, or symmetric SMCV of both sides deteriorated as ipsilateral delayed SMCV in follow-up 4D-DSA imaging.

### Radiological and clinical assessment

A threshold delay time (DT) of >3 s was used for volumetric measurement of baseline and follow-up hypoperfusion area. Relative cerebral blood flow (rCBF) <30% within the DT > 3 s was used for calculating baseline ischemic core volume (8). DT was calculated by a modified singular value deconvolution approach by looping through a series of DT values (9). For 3–5 days, DWI or NCCT was used for calculating the final infarct volume. DWI lesions were assessed with a b value of 1,000 s/mm^2^. Volumetric analysis was performed using MIStar software. The baseline mismatch ratio was defined as baseline hypoperfusion volume divided by baseline ischemic core volume. Collateral index was defined as DT > 6 s divided by DT > 2 s^*^100% (10). ^***^Reperfusion rate = (baseline hypoperfusion volume- follow-up hypoperfusion volume)/baseline hypoperfusion volume. We defined the two groups as reperfusion (≥80%) and no reperfusion (<80%) (5). Furthermore, we assessed the reperfusion status using modified thrombolysis in cerebral infarction (mTICI) scores in the subgroup of patients who received thrombectomy and defined an mTICI score of 2b-3 as successful reperfusion (11, 12). Hemorrhagic transformation (HT) was classified as hemorrhagic infarction (HI) or parenchymal hemorrhage (PH), according to the definition of the European Cooperative Acute Stroke Study (ECASS) (13). The mRS score was used to identify the clinical outcome at 3 months. An mRS score of 0–2 was designated a good outcome, and an mRS score of 3–6 was designated a poor outcome.

Two people (JSX and ZYZ. with 2 years of experience with neuroimaging analysis) rated and jointly evaluated the imaging markers. The raters were blinded to the patients' 24-h imaging and clinical data. A single trained observer (JSX) measured imaging markers in all patients twice, at an interval of 1 month apart. Another observer (ZYZ) independently made the same evaluation. If the two raters had different diagnosis opinions, the result was decided by a senior neuroimaging expert (SZ, with 10 years of experience in neuroimaging analysis).

### Statistical analysis

Two raters (JSX and ZYZ) who jointly evaluated the images of SMCV and HT were blinded to the patients' other imaging and clinical data. A single trained observer (SZ) reviewed all data two times, at an interval of 3 months apart. Another observer (JSX) independently made the same evaluation.

Cohen's kappa coefficient was used to assess the level of inter- and intra-observer agreement for evaluating SMCV filling deficit on both hemispheres, HT and PH. Excellent inter- and intra-observer agreement was seen in distinguishing baseline delayed SMCV (κ = 0.792 and 0.854), baseline SMCV-(κ = 0.893 and 0.835), follow-up SMCV status (κ = 0.809 and 0.844), HT (κ = 0.962 and 0.887), and PH (κ = 1.000 and 1.000).

All numeric variables were expressed as mean ± standard deviation (SD) and median (interquartile range, IQR). Categorical variables were presented as frequency (percentage). Fisher's exact test was used to compare dichotomous variables between groups, while the Mann–Whitney *U* test was used for ordered categorical variables. An independent sample two-tailed *t*-test or Mann–Whitney *U* test was used for continuous variables, depending on the normality of the distribution. Variables identified by univariable analysis (*p* < 0.05) were included in the binary logistic regression model. All analyses were performed with blinding of the participants' identification information. Statistical analysis was performed using SPSS, version 19.0 (IBM, Armonk, NY).

## Results

A total of 54 patients were enrolled in this analysis, including 19 female patients (35.2%) with a median age of 69 years (IQR: 56–81 years) and a median NIHSS score of 14 (IQR: 8–18). A total of 44 patients received reperfusion therapy, including 13 patients (24.1%) who received intravenous thrombolysis and 37 patients (68.5%) who received EVT (four patients received bridging therapy).

### The association between reperfusion and SMCV reversal

Of 54 patients with LVO, seven patients were shown as symmetric SMCV and 47 (87.0%) as delayed SMCV at baseline (including 27.7% (13/47) SMCV-), with an average of 7.5 ± 6.9 s in time delay. Of the 54 patients, all seven symmetric SMCVs at baseline were still symmetric in follow-up dynamic 4D-DSA imaging, while in patients who showed delayed SMCV at baseline (*n* = 47), 36 patients (76.6%) were reversed as symmetric SMCV (SMCV reversal) and 11 patients (23.4%) had persistent delayed SMCV on follow-up dynamic 4D-DSA imaging. Among these 11 patients with persistent delayed SMCV, six received EVT, including three who showed reocclusion or restenosis with repeated hypoperfusion according to the follow-up CTP imaging (reperfusion failure), while the remaining three patients (3/54, 5.6%) showed persistent SMCV- on both baseline and follow-up four-dimensional CTA (4D-CTA).

Baseline delayed SMCV was associated with the baseline volume of DT > 2 s and hypoperfusion (DT > 3 s) (both *p* < 0.05), but not associated with baseline collateral index (*p* > 0.05). Among the patients who showed baseline delayed SMCV (*n* = 47), univariable analysis showed that baseline collateral index, baseline mismatch ratio, and reperfusion were associated with SMCV reversal (both *p* < 0.05) (Table 1). Multivariable regression analysis showed that reperfusion (odds ratio (OR) = 69.328, 95% confidence interval (CI) = 2.818–175.342, *p* = 0.009) was independently associated with SMCV reversal after adjusting for baseline mismatch ratio (OR = 0.920, 95%CI = 0.863–0.981, *p* = 0.008) and collateral index (OR = 1.075, 95%CI = 0.961–1.203, *p* = 0.206).

**Table 1:** Baseline and follow-up characteristics of patients stratified by the SMCV status.

**Baseline SMCV status**	**Symmetric SMCV (*n =* 7)**	**Delayed SMCV (** ***n =*** **47)**		**Test value**	***P* value**	**Test value^a^**	***P* value^a^**
**Follow-up SMCV status**	**Symmetric SMCV (*n =* 7)**	**Reversed SMCV^a^** **(*n =* 36)**	**Sustained delayed SMCV^a^ (*n =* 11)**				
Age (years), median (IQR)	79 (75–81)	68.5 (56.3–81.8)	63 (47–81)	F = 1.359	0.266	t = −0.693	0.492
Female, *n* (%)	2 (28.6)	14 (38.9)	3 (27.3)	χ^2^ = 0.653	0.722	χ^2^ = 0.492	0.483
Time gap of SMCV opacification in both sides (seconds), median (IQR)	0 (0–0)	8.0 (3.9–11.8)	7 (2–13)	F = 6.662	0.003	t = −1.181	0.244
Onset to door time, hours, median (IQR)	8.5 (4–13)	7.8 (4–11)	7 (2–13)	F = 0.082	0.922	Z = −0.769	0.449
Onset to reperfusion time, hours, median (IQR)^b^	6 (5.5–15.5)	9 (4–12)	9 (6–10)	F = 0.025	0.976	Z = −0.136	0.912
Occlusion site, *n* (%)				χ^2^ = 9.174	0.057	χ^2^ = 5.362	0.068
MCA-M1 segment	7 (100)	26 (72.2)	6 (54.5)				
ICA	0 (0)	3 (8.3)	4 (36.1)				
ICA+M1	0 (0)	7 (19.4)	1 (9.1)				
Baseline NIHSS score, median (IQR)	12 (7–18)	14.5 (9–19.8)	10 (6–18)	F = 0.568	0.570	Z = −0.944	0.351
LAA, *n* (%)	3 (42.9)	23 (63.9)	9 (81.8)	χ^2^ = 2.888	0.346	χ^2^ = 1.246	0.264
Cardiogenic stroke, CS, *n* (%)	4 (57.1)	13 (36.1)	2 (18.2)	-	-	-	-
Medical history							
Hypertension, *n* (%)	6 (85.7)	22 (61.1)	9 (81.8)	χ^2^ = 2.777	0.249	χ^2^ = 1.609	0.205
Diabetes mellitus, *n* (%)	2 (28.6)	5 (13.9)	2 (18.2)	χ^2^ = 0.932	0.627	χ^2^ = 0.123	0.726
Atrial fibrillation, *n* (%)	3 (42.9)	12 (33.3)	3 (27.3)	χ^2^ = 0.468	0.792	χ^2^ = 0.142	0.706
Previous TIA/ Stroke, *n* (%)	1 (14.3)	3 (8.3)	1 (91.)	χ^2^ = 0.248	0.884	χ^2^ = 0.006	0.937
Baseline systolic blood pressure (mmHg), mean ± SD	169.1 ± 16.6	153.7 ± 28.0	153.6 ± 26.8	F = 1.023	0.367	t = −0.006	0.995
Baseline diastolic blood pressure (mmHg), mean ± SD	92.4 ± 17.6	87.9 ± 13.9	86.3 ± 26.2	F = 0.279	0.757	t = −0.270	0.789
Baseline serum glucose, mmol/L, mean ± SD	8.8 ± 5.6	7.0 ± 1.6	7.3 ± 2.5	F = 1.530	0.226	t = 0.470	0.641
Collateral index (%), mean ± SD	14.5 ± 12.0	16.4 ± 12.9	8.3 ± 14.0	F = 1.610	0.210	t = 0.748	0.082
Baseline ischemic core volume, ml, mean ± SD	16.1 ± 17.7	18.1 ± 17.3	12.8 ± 24.3	F = 0.334	0.718	t = −0.805	0.425
Baseline hypoperfusion volume, ml, mean ± SD	64.1 ± 53.8	98.9 ± 60.6	117.8 ± 88.6	F = 1.407	0.254	t = 0.808	0.423
Baseline mismatch ratio, mean ± SD	6.8 ± 5.9	10.4 ± 13.0	44.6 ± 35.6	F = 14.522	<0.001	t = 4.893	<0.001
Delay time > 2 s, ml, mean ± SD	90.6 ± 54.6	137.6 ± 78.3	188.4 ± 104.6	F = 3.198	0.049	t = 1.737	0.089
Delay time > 6s, ml, mean ± SD	12.6 ± 13.9	27.2 ± 30.1	24.1 ± 50.5	F = 0.547	0.582	t = −0.247	0.806
Intravenous thrombolysis, *n* (%)	3 (42.9)	7 (19.4)	3 (27.3)	χ^2^ = 1.835	0.400	χ^2^ = 0.308	0.579
Thrombectomy, *n* (%)	3 (42.9)	28 (77.8)	6 (54.5)	χ^2^ = 4.563	0.102	χ^2^ = 2.273	0.132
Reperfusion, *n* (%)	4 (57.1)	30 (83.3)	3 (27.3)	χ^2^ = 3.596	0.058	χ^2^ = 12.661	<0.001
NIHSS at discharge, median (IQR)	3 (1–15)	4.5 (2–12)	9 (4–19)	F = 2.215	0.120	Z = −1.801	0.072
Final infarct core volume, ml, mean ± SD	12.4 ± 9.7	41.9 ± 42.0	89.1 ± 93.3	F = 4.911	0.011	Z = −1,018	0.314
Infarct growth volume, ml, median (IQR)	−3.7 ± 14.9	25.9 ± 33.9	76.3 ± 85.3	F = 6.783	0.003	Z = −1.754	0.082
HT, *n* (%)	2 (28.6)	20 (55.6)	2 (18.2)	χ^2^ = 5.587	0.061	χ^2^ = 4.727	0.030
PH, *n* (%)	0 (0)	4 (11.1)	0 (0)	χ^2^ = 2.160	0.340	χ^2^ = 1.336	0.248

^a^Indicates the comparison between reversed SMCV (*n* = 36) and sustained delayed SMCV (*n* = 11) in patients with baseline delayed SMCV (*n* = 47).

^b^Indicates that these data were only available for patients who received endovascular therapy (EVT) (*n* = 37).

SMCV, superficial middle cerebral vein; SMCV-, absent filling of the ipsilateral superficial middle cerebral vein; IQR, interquartile range; NIHSS, National Institute of Health stroke scale; LAA, large artery atherosclerosis; TIA, transient ischemic attack; SD, standard deviation; HT, hemorrhagic transformation; PH, parenchymal hemorrhage.

### The association between SMCV reversal and neurological outcome

Univariable analysis showed that SMCV reversal was not significantly associated with final infarct volume, infarct growth, NIHSS at discharge, and a 3-month poor outcome (all values of *p* > 0.05).

Superficial middle cerebral vein reversal was associated with a higher rate of HT (55.6 vs. 18.2%, χ^2^ = 4.747, *p* = 0.030), and all PHs (100%, 4/4) were found in the SMCV reversion group. Binary logistic regression analysis showed that SMCV reversal had an approximately 6-fold risk of HT than persistent delayed SMCV (OR = 5.714, 95% CI = 1.041–31.357, *p* = 0.045). While this effect of SMCV reversal (OR = 1.747, 95% CI = 0.351–8.683, *p* = 0.495) was rectified after adjusting for reperfusion.

Further, a 3-month poor outcome was associated with DT > 6 s volume, baseline SMCV delay, and reperfusion (all values of *p* < 0.05). Univariable comparison analysis between patients with good and poor outcomes is shown in Table 2. In multivariable regression analysis, baseline delayed SMCV was positively associated with a poor outcome (OR = 19.623, 95% CI = 1.567–245.727, *p* = 0.021), while reperfusion was negatively associated with a poor outcome (OR = 0.131, 95% CI = 0.025–0.686, *p* = 0.016).

**Table 2:** Comparisons of baseline and follow-up characteristics between patients with a good and poor outcome at 3 months.

	**Good outcome (*n =* 27)**	**Poor outcome (*n =* 27)**	**Test value**	***P* value**
Age (years), median [IQR]	68 (56–80)	73 (57–82)	Z = −0.753	0.451
Female, *n* (%)	8 (29.6)	11 (28.6)	χ^2^ = 0.731	0.393
Baseline NIHSS score, median (IQR)	12 (8–17)	14 (9–19)	Z = −0.641	0.522
Onset to door time, hours, median (IQR)	7.5 (4–11)	8 (4–13)	Z = −0.781	0.435
Onset to reperfusion time, hours, median (IQR)^a^	9 (5.8–10.8)	9 (6–10)	Z = −0.031	0.975
LAA, *n* (%)	19 (70.4)	16 (59.3)	χ^2^ = 0.731	0.393
Previous history				
Hypertension, *n* (%)	20 (74.1)	17 (63.0)	χ^2^ = 0.773	0.379
Diabetes mellitus, *n* (%)	6 (22.2)	3 (11.1)	χ^2^ = 1.200	0.273
Atrial fibrillation, *n* (%)	6 (22.2)	12 (44.4)	χ^2^ = 3.000	0.074
Previous TIA/ Stroke, *n* (%)	2 (7.4)	3 (11.1)	χ^2^ = 0.220	0.639
Occlusion site, *n* (%)			χ^2^ = 0.668	0.716
MCA–M1 segment	19 (70.4)	20 (74.1)		
ICA	3 (11.1)	4 (14.8)		
ICA+M1	5 (18.5)	3 (11.1)		
Baseline systolic blood pressure (mmHg), mean ± SD	151 (131–182)	159 (142–175)	Z = −0.216	0.829
Baseline diastolic blood pressure (mmHg), mean ± SD	88 (78–105)	80 (73–96)	Z = −1.341	0.180
Baseline serum glucose (mmol/L), mean ± SD	7.7 ± 3.1	7.0 ± 2.0	Z = −1.410	0.882
Baseline ischemic core volume (ml), median (IQR)	9 (4–22)	11 (3–20)	Z = −0.113	0.910
Baseline hypoperfusion volume (ml), median (IQR)	88 (52–155)	83 (51–112)	Z = −0.900	0.368
Baseline mismatch ratio, median (IQR)	6.6 (4.4–19.2)	5.9 (3.2–12.3)	Z = −0.770	0.441
Delay time > 2s (ml), Median (IQR)	139 (95–200)	126 (89–145)	Z = −1.419	0.156
Delay time > 6s(ml), Median (IQR)	16 (5–55)	9 (0–23)	Z = −2.013	0.044
Collateral index, mean±SD	0.29 ± 0.63	0.36 ± 0.91	Z = −1.773	0.076
Baseline SMCV delay, *n* (%)	21 (77.8)	26 (96.3)	χ^2^ = 4.103	0.043
Baseline SMCV-, *n* (%)	5 (18.5)	8 (29.6)	χ^2^ = 0.912	0.340
Follow-up SMCV filling, *n* (%)				
Symmetric SMCV	6 (22.2)	1 (3.7)	χ^2 =^ 5.844	0.019
Reversed SMCV	18 (66.7)	18 (66.7)		
Sustained delayed SMCV	3 (11.1)	8 (29.6)		
Intravenous thrombolysis, *n* (%)	8 (29.6)	5 (18.5)	χ^2^ = 0.912	0.340
Thrombectomy, *n* (%)	21 (77.8)	16 (59.3)	χ^2^ = 2.146	0.143
Reperfusion, *n* (%)	23 (85.2)	14 (51.9)	χ^2^ = 6.954	0.008
NIHSS at discharge, median (IQR)	3 (1–5)	11 (5–18)	Z = −3.792	<0.001
Final infarct core volume, ml, median (IQR)	12 (7–27)	36 (20–85)	Z = −3.400	0.001
Infarct growth volume, ml, median (IQR)	6 (−6–26)	29 (13–84)	Z = −3.049	0.002
HT, *n* (%)	10 (37.0)	14 (51.9)	χ^2^ = 1.200	0.273
PH, *n* (%)	1 (3.7)	3 (11.1)	χ^2^ = 1.080	0.299

^a^Indicates that these data were only available for patients who received EVT (*n* = 37).

SMCV, superficial middle cerebral vein; SMCV-, absent filling of the ipsilateral superficial middle cerebral vein; IQR, interquartile range; NIHSS, National Institute of Health stroke scale; LAA, large artery atherosclerosis; TIA, transient ischemic attack; SD, standard deviation; TICI, Thrombolysis in Cerebral Infarction; HT, hemorrhagic transformation; PH, parenchymal hemorrhage.

**Figure 2:**
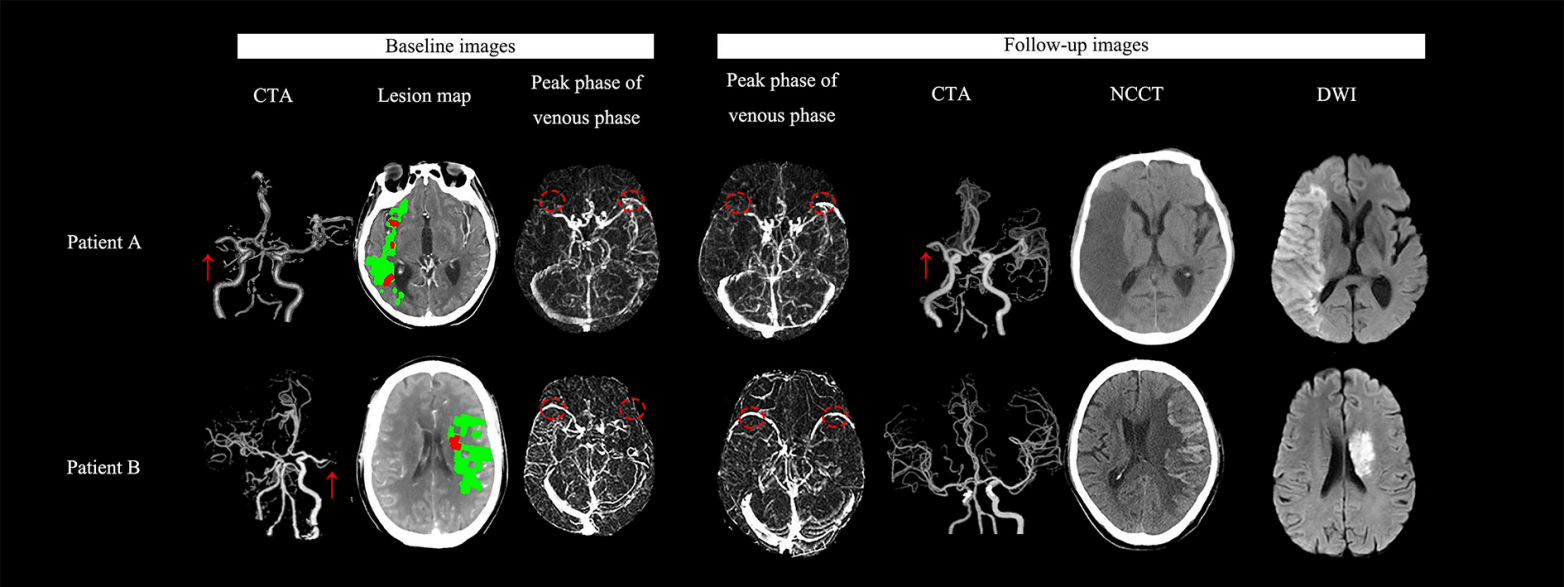
Examples for illustrating the association between the stroke outcome and the dynamic change of SMCV filling. **(Patient A)** A 63-year-old man with right cerebral middle artery (R-MCA) occlusion (red arrow) presented with 7-ml baseline ischemic core volume (red color area on the lesion map) and ipsilateral delayed SMCV (red circle) on auto-reconstructed four-dimensional computed tomographic angiography (4D-CTA) using MIStar software. He received thrombectomy after onset. On follow-up 4D-CTA, delayed SMCV was still presented. We did not find hemorrhagic transformation (HT) on his follow-up NCCT scans. His final infarct volume (FIV) was 161 ml. His National Institutes of Health Stroke Scale (NIHSS) score was 21 and modified Rankin scale (mRS) score at discharge and 3 months were 5 and 4 respectively. **(Patient B)** A 65-year-old man with acute left middle cerebral artery (L-MCA) occlusion (red arrow) presented with 3-ml baseline ischemic core volume (red color area on the lesion map) and ipsilateral delayed SMCV (red circle) on 4D-CTA. He did not receive any reperfusion therapy but achieved a spontaneous recanalization. On follow-up 4D-CTA, delayed SMCV reversed to be bilaterally symmetric. On 24 h NCCT after admission, we did not find HT. His FIV was 27 ml. His NIHSS score was 24, and the mRS score at discharge and 3 months were 4 and 4 respectively.

When follow-up SMCV status was enrolled into the regression model, reperfusion was still negatively associated with a poor outcome (OR = 0.122, 95%CI = 0.018–0.826, *p* = 0.031); patients with SMCV reversal (OR = 21.035, 95%CI = 1.413–113.250, *P* =0.027) and persistent SMCV delay (OR = 17.876, 95%CI = 1.093–292.491, *p* = 0.043) had nearly 21- and 17-fold risk of a 3-month poor outcome, separately, in comparison with patients with symmetric SMCV. No significant difference was noted in the risk of a poor outcome between patients with SMCV reversal and persistent SMCV delay (OR = 1.177, 95%CI = 0.147–9.448, *p* = 0.878) (Figure 2).

## Discussion

This study is the first to use the ipsilateral delayed SMCV filling method to distinguish stroke-related venous abnormalities and congenital venous variations, and we revealed the significance of delayed SMCV filling for reperfusion therapy and stroke prognosis. We found that baseline ipsilateral delayed SMCV was associated with the degree of baseline hypoperfusion volume and delayed SMCV can be reversed to symmetric when reperfusion was successfully achieved, indicating that the changes in SMCV filling were related to the tissue perfusion status. However, SMCV reversal cannot significantly reverse the stroke prognosis, which was related to the status of baseline SMCV filling and reperfusion, supporting that SMCV reversal was only a marker of reperfusion rather than a factor affecting prognosis, more attention should be paid to baseline SMCV filling and more active reperfusion therapy should be recommended for improving the stroke outcome.

Our previous research found the phenomenon of SMCV reversal (5); however, the formation mechanism of SMCV filling deficit is still unclear. Reportedly, the occluded cerebral veins returned to normal after anticoagulant treatment in patients with venous sinus thrombosis (14). In our patients with LVO, delayed SMCV filling can be reversed to normal through reperfusion after thrombolysis or thrombectomy but not through anticoagulant treatment. This result indicated that delayed SMCV might develop due to blood stasis or reduced blood flow that can be drained but not because of the occlusion of venous thrombosis.

Notably, SMCV reversal did not go concurrently with the achievement of reperfusion. In our study, six patients showed delayed or absent filling of SMCV in baseline and follow-up 4D-CTA. These patients received thrombectomy and achieved recanalization initially, including five patients who reached TICI 3 and one patient who reached TICI 2b. However, according to the follow-up reconstructed 4D-CTA (3–5 days after thrombectomy), three of them had vascular reocclusion or restenosis, resulting in repeat hypoperfusion of the previous ischemic brain tissue (reperfusion failure). The other three patients (3/54, 5.6%) showed persistent absent filling of SMCV from baseline to the follow-up, including one patient with acute left MCA occlusion and two with right ICA occlusion. This may be due to the anatomical congenital variation of SMCV. It was shown that 82% of healthy people have bilateral symmetrical SMCV, approximately 2% showed right-side dominant laterality, and 16% showed left-side dominant laterality (15). In this case, if the non-dominant side of SMCV is too thin, 4D-CTA reconstructed according to CTP may not be displayed. Therefore, the reocclusion phenomenon and congenital variation of SMCV both contributed to the result that the reversal of SMCV was not completely consistent with the reperfusion state.

This mechanism of the formation of delayed SMCV was only speculated based on the structural opacification of SMCV on 4D-DSA, which may be limited in its ability to reflect venous drainage function. In SWI studies, Xia et al. reported that the prominent cortical vein on the affected side was significantly associated with low blood oxygen saturation (4), and Seyedmehdi et al. reported that the absence of cortical veins was significantly correlated with large artery occlusion and infarct volume on SWI (3). However, there is no study yet to confirm the relationship between the low blood oxygen saturation represented by the prominent cortical vein and delayed SMCV opacification. A recent study showed that improved oxygen saturation of asymmetric cortical veins detected using brain SWI with quantitative susceptibility mapping (QSM) corresponded with better acute ischemic stroke outcomes for patients with asymmetrically prominent cortical veins that disappeared in the 2-week follow-up magnetic resonance imaging (MRI) (16). This result inspired us to dynamically evaluate the venous drainage in combination with venous structure and function in the future for a better illustration of the formation mechanism of abnormal SMCV filling.

By using prospectively collected INSPIRE data, we proved that baseline delayed SMCV was closely related to long-term stroke outcome, while SMCV reversal failed to reverse this effect. This result was consistent with our previous finding that (4, 17) baseline SMCV- was a poor predictor of the stroke outcome in patients who received intravenous thrombolysis, and SMCV- patients that reversed to normal at 24 h showed no difference in clinical outcomes in comparison to those with persistent SMCV- at 24 h. In this study, we further found that even delayed SMCV (a more subtle change of SMCV filling deficit than SMCV-) at baseline was associated with the stroke outcome in LVO. Patients with reversed SMCV still showed a higher risk of poor outcomes compared to patients with persistent symmetric SMCV, indicating that although reperfusion improved the hemodynamics of ipsilateral SMCV, such a recovery can either subsequently bring a better outcome than persistent delayed SMCV or reverse the poor stroke prognosis. The potential explanations for this result include the following: (1) In the case of acute LVO, baseline delayed SMCV may reflect a hemodynamic change, such as the speed at which collateral blood flows out of the venous side. After reperfusion, even if SMCV opacification returns to symmetrical, it can only reflect that the blood flow on the arterial side can flow smoothly from the venous side but cannot reflect the utilization of cerebral blood flow by the brain tissue. This has been confirmed from our baseline data analysis in which there was no significant correlation between the venous reflux level and the tissue collateral level (the correlation coefficient between baseline delayed SMCV and a collateral index did not reach statistical significance). (2) Because most patients have baseline delayed SMCV after acute LVO, even if the SMCV returns to symmetry after reperfusion, the probability of edema and PH in patients is higher than that in patients who have symmetrical bilateral SMCV at baseline (6, 7). This reperfusion injury perhaps offset the benefits brought by the restoration of symmetry of SMCV. On the other hand, we can also conclude that it may be enough to predict the stroke outcome by observing the baseline opacification status of SMCV. Although follow-up SMCV status was associated with a risk of poor stroke prognosis, it did not provide extra value or enhance the predictive ability of baseline delayed SMCV.

The advantage of this study was that we used a simple method to evaluate SMCV. We adopted ipsilateral delayed SMCV as a more hemodynamic evaluation method to better reflect that the filling deficit of SMCV was caused by cerebral ischemia. This method can efficiently find out whether patients have congenital dysplasia or variation and capture the relationship between the filling deficit of SMCV and hypoperfusion more sensitively. In addition to that, now that the predictive value of baseline delayed SMCV was repeatedly proven, there is no need to evaluate the filling of SMCV on tMIP function and CTP-derived 4D-CTA in further studies, as the cortical veins frequently start to present at the peak arterial phase. Consequently, we can identify patients with delayed SMCV if there is a poor opacification of ipsilateral SMCV on brain single-phase CTA, which reflects the vascular opacification at the peak arterial phase.

Interestingly, in 17 patients who did not receive successful reperfusion (mTICI <2b), baseline delayed SMCV in six patients reversed to bilateral symmetry without receiving any reperfusion therapy, indicating that (1) SMCV filling did not particularly depend on the degree of reperfusion or (2) the SMCV filling may depend on other cerebral hemodynamic characteristics in addition to the reperfusion rate. This is worthy of further exploration in the future to better explain the mechanism of the SMCV filling deficit.

## Limitations

This study had limitations. Firstly, this was a retrospective study with a small sample size. Secondly, as the follow-up CT perfusion was not a regular test for our patients, and some patients who had severe neurological deficits were unable to stand for the follow-up CT perfusion examination, we were unable to evaluate their reperfusion status. Thirdly, in our study, patients with and without reperfusion therapy were mixed. Due to the limitation of the sample size, we were unable to observe the effect of SMCV changes on outcomes in each subgroup. However, we have confirmed the effect of reperfusion on SMCV, and we will further quantify the opacification of SMCV in a larger sample population in the future. Finally, most of our readings were based on 4D-DSA images automatically generated by MIStar software to interpret the opacification of SMCV, which was not objective for some patients with a head tilt who were also vulnerable to the influence of layer thickness. Although we reanalyzed the original images of the samples to reduce the probability of the above mentioned misjudgments, it is also where MIStar software can contribute in the future, by adding automatic head position calibration or providing a thinner slice display.

## Conclusion

Delayed SMCV and SMCV reversal, as the outlet of arterial and microcirculatory levels, were the result of hypoperfusion and reperfusion, separately. No matter whether the baseline delayed SMCV returns to symmetrical or non-symmetrical results, baseline delayed SMCV is an independent cause of poor stroke prognosis in patients with LVO. The evaluation of baseline SMCV filling status should be strengthened in clinical practice.

## Data availability statement

The raw data supporting the conclusions of this article will be made available by the authors, without undue reservation.

## Ethics statement

The studies involving human participants were reviewed and approved by human Ethics Committee of Zhejiang Provincial People's Hospital. The patients/participants provided their written informed consent to participate in this study.

## Author contributions

Study conceptualization and design and manuscript development: JX and SZ. Literature review: BJ and YG. Data analysis and interpretation of results: ZZ, SZ, and LL. All authors contributed to the article and approved the submitted version.

## Funding

This work was supported by the Zhejiang Provincial Natural Science Foundation of China (Grant No. LGF22H090020), the Medical Health Science and Technology Project of the Zhejiang Provincial Health Commission (Grant No. 2022KY600), and Key project of the Department of Science and Technology of Zhejiang Province (2018C03008). The perfusion analysis software (MIStar) was provided to the site as part of their involvement in the INSPIRE (www.Inspire.apollomit.com/), a study funded by the National Health and Medical Research Council of Australia.

## Conflict of interest

The authors declare that the research was conducted in the absence of any commercial or financial relationships that could be construed as a potential conflict of interest.

## Publisher's note

All claims expressed in this article are solely those of the authors and do not necessarily represent those of their affiliated organizations, or those of the publisher, the editors and the reviewers. Any product that may be evaluated in this article, or claim that may be made by its manufacturer, is not guaranteed or endorsed by the publisher.
